# Cachexia causes time‐dependent activation of the inflammasome in the liver

**DOI:** 10.1002/jcsm.13236

**Published:** 2023-05-12

**Authors:** Rodrigo Xavier das Neves, Alex S. Yamashita, Daniela M.R. Riccardi, Julia Köhn‐Gaone, Rodolfo G. Camargo, Nelson I. Neto, Daniela Caetano, Silvio P. Gomes, Felipe H. Santos, Joanna D.C.C. Lima, Miguel L. Batista, José Cesar Rosa‐Neto, Paulo Sérgio Martins De Alcântara, Linda F. Maximiano, José P. Otoch, Giorgio Trinchieri, Janina E.E. Tirnitz‐Parker, Marília Seelaender

**Affiliations:** ^1^ Cancer Metabolism Research Group, Department of Surgery and LIM26‐HCFMUSP Faculdade de Medicina University of São Paulo São Paulo Brazil; ^2^ LICI, Center for Cancer Research National Cancer Institute Bethesda MD USA; ^3^ Department of Physiology and Biophysics, Institute of Biomedical Sciences University of São Paulo São Paulo Brazil; ^4^ Department of Surgery School of Veterinary Medicine and Animal Science of University of São Paulo—FMVZ/USP São Paulo Brazil; ^5^ Department of Physiology Federal University of São Paulo São Paulo Brazil; ^6^ Laboratory of Adipose Tissue Biology, Center for Integrated Biotechnology University of Mogi das Cruzes São Paulo Brazil; ^7^ Immunometabolism Research Group, Department of Cell and Developmental Biology, Institute of Biomedical Sciences University of São Paulo São Paulo Brazil; ^8^ Liver Disease and Regeneration Laboratory, School of Pharmacy and Biomedical Sciences and Curtin Health Innovation Research Institute Curtin University Bentley Western Australia Australia

**Keywords:** cancer cachexia, inflammasome, inflammation, liver, myeloid cells

## Abstract

**Background:**

Cachexia is a wasting syndrome associated with systemic inflammation and metabolic disruption. Detection of the early signs of the disease may contribute to the effective attenuation of associated symptoms. Despite playing a central role in the control of metabolism and inflammation, the liver has received little attention in cachexia. We previously described relevant disruption of metabolic pathways in the organ in an animal model of cachexia, and herein, we adopt the same model to investigate temporal onset of inflammation in the liver. The aim was thus to study inflammation in rodent liver in the well‐characterized cachexia model of Walker 256 carcinosarcoma and, in addition, to describe inflammatory alterations in the liver of one cachectic colon cancer patient, as compared to one control and one weight‐stable cancer patient.

**Methods:**

Colon cancer patients (one weight stable [WSC] and one cachectic [CC]) and one patient undergoing surgery for cholelithiasis (control, *n* = 1) were enrolled in the study, after obtainment of fully informed consent. Eight‐week‐old male rats were subcutaneously inoculated with a Walker 256 carcinosarcoma cell suspension (2 × 10^7^ cells in 1.0 mL; tumour‐bearing [T]; or phosphate‐buffered saline—controls [C]). The liver was excised on Days 0 (*n* = 5), 7 (*n* = 5) and 14 (*n* = 5) after tumour cell injection.

**Results:**

In rodent cachexia, we found progressively higher numbers of CD68^+^ myeloid cells in the liver along cancer‐cachexia development. Similar findings are described for CC, whose liver showed infiltration of the same cell type, compared with both WSC and control patient organs. In advanced rodent cachexia, hepatic phosphorylated c‐Jun N‐terminal kinase protein content and the inflammasome pathway protein expression were increased in relation to baseline (*P* < 0.05). These changes were accompanied by augmented expression of the active interleukin‐1β (IL‐1β) form (*P* < 0.05 for both circulating and hepatic content).

**Conclusions:**

The results show that cancer cachexia is associated with an increase in the number of myeloid cells in rodent and human liver and with modulation of hepatic inflammasome pathway. The latter contributes to the aggravation of systemic inflammation, through increased release of IL‐1β.

## Introduction

Cancer cachexia is a devastating syndrome, severely reducing the quality of life and overall survival in cancer patients.^1^ It represents the direct cause of death for ~20–40% associated with the disease.^2^ It is considered a multifactorial syndrome, characterized by rapid and progressive involuntary weight loss, sustained by both adipose tissue depletion and muscle wasting, which cannot be reversed by conventional nutrition therapy.^3^

Cachectic patients present systemic chronic inflammation.^2^ Nevertheless, the compartment(s) triggering the release of pro‐inflammatory cytokines remain(s) not fully established, as this alteration probably results from an orchestration of signals deriving from the tumour, host organs and immune system cells.^4^ Many studies have shown that the visceral organs are able to markedly contribute to the increase pro‐inflammatory cytokines expression, which could sustain or augment cachexia.^5^

The liver is the central organ in the control of energy substrate distribution to the organism and governs intermediary metabolism, regulating the transport, storage, breakdown and utilization of glucose, lipids and proteins.^6^ Impairment of liver metabolism during cachexia has been found to favour disease progression, by causing energy imbalance in the face of compromised mitochondrial function.^7^, ^8^ Despite the fact that many of the disruptions present in the liver in cachexia could bear a relevant relationship with muscle wasting,^9^ much remains to be addressed.

In this study, we investigated whether the inflammasome and nuclear factor‐κB (NF‐κB) pathways are activated in the liver of cachectic tumour‐bearing rats, having shown before, in the same model, that inhibition of prostaglandin E_2_ (PGE_2_) synthesis ameliorates cachexia, with a direct effect in the liver.^10^, ^11^ According to Martignoni et al.,^12^ the common myeloid marker CD68 is increased in the livers of cachectic patients, compared to non‐cachectic counterparts. These cells are associated with a more aggressive tumour phenotype.^12^ We thus wanted to address the hypothesis that the same would be observed in the well‐established Walker 256 model of cachexia and in the liver of cachectic patients with colon cancer, the third most common cancer in the world.^13^ Furthermore, as inflammatory cell infiltration has been robustly associated with increased inflammatory cytokine expression and secretion in many organs, including the liver,^14^ we examined cytokine secretion and the respective intracellular pathways involved in their production.

Finally, as to confirm that the alterations were not exclusive to rodent cachexia, we examined the liver of two colon cancer patients (cachectic and weight stable) in comparison to a patient who did not have cancer.

## Materials and methods

### Casuistics

Liver biopsies from two colon cancer patients and one non‐cancer patient (cholelithiasis) obtained during surgery were analysed. The two patients with colon cancer were classified as (1) weight stable (WSC) and (2) cachectic (CC). The WSC patient did not present weight loss in the last 6 months, and his body mass index (BMI) was 26.0, C‐reactive protein concentration was 3.7 mg/L and albumin concentration was 5.63 g/dL. The CC patient showed weight loss of 29.1% within the 6 months prior to the enrolment, with BMI of 20.7, C‐reactive protein concentration of 12.1 mg/L and albumin concentration equal to 2.55 g/dL. The non‐cancer patient did not report any weight loss in the previous semester and presented with a BMI of 24.65, C‐reactive protein concentration equal to 0.2 mg/L and the circulating albumin concentration of 4.41 g/dL. The study was carried out in accordance with the recommendations of the Ethics Committee on Research involving Human Subjects of the Biomedical Sciences Institute of the University of São Paulo (USP) (1117/CEP) and was also approved by the Human Ethics Committee of the University Hospital/USP (CEP 1388/14). The study was registered in the Brazilian authority platform under Number CAEE:14197413.9.0000.5467. Written informed consent was obtained from all the patients before admission to the protocol.

### Animals

Male adult Wistar rats (270–300 g), obtained from the Institute of Biomedical Sciences, USP, were maintained in metabolic cages, under a 12‐h light/12‐h dark cycle and controlled temperature conditions (23 ± 1°C), receiving water and food (commercial chow; Nuvilab, Nuvital, Brazil) ad libitum. The Institute of Biomedical Sciences/USP Ethical Committee for Animal Research approved all the adopted procedures (050/102 ECAR), which were carried out in accordance with the ethical principles stated by the Brazilian College of Animal Experimentation. A Walker 256 tumour cell suspension (2 × 10^7^ cells) was injected subcutaneously into the right flank of the animals.^12^ Control rats (*n* = 5) received saline injections on the same day of tumour inoculation. The experiments were designed as a time‐course study, and the rats were sacrificed by decapitation on Days 0 (baseline), 7 and 14 after inoculation (*n* = 5 per group) following a 12‐h fasting period.

### Patient liver collection

#### Obtainment of liver biopsies

During surgery, a 0.5‐cm^3^ fragment of the liver was collected, without interfering with regular surgical procedure (the biopsy was for the assessment of metastasis in the case of the cancer patients, whereas in the control patient, also the normal procedure was strictly followed). The tissues were transferred to 4% paraformaldehyde for fixation and then embedded in paraffin; non‐serial 5‐μm sections were obtained for histological evaluation.

### Rodent study

The livers were excised and weighed, and one fragment of 0.5 cm was embedded in optimal cutting temperature (OCT) compound for histological analyses. The remainder was snap‐frozen in liquid nitrogen and stored at −80°C for RNA and protein analyses.

### Immunohistochemistry

The livers were placed into plastic cassettes, mounted with OCT compound medium (Sakura Finetek Japan Co., Ltd., Tokyo, Japan) and frozen in liquid nitrogen. Sections were obtained (7 μm) and subjected to haematoxylin and eosin staining for the analysis of liver histology as well as for immunohistochemical study. Liver sections were fixed with methanol/acetone (1:1), permeabilized with 0.02% Tween 20 in phosphate‐buffered saline (PBS‐T) and blocked with 5% goat serum in PBS‐T. Sections were incubated overnight at 4°C with anti‐CD68 (1:200, Abcam, ab 31630, rabbit) to detect liver macrophages. Antibody detection was performed by employing a horseradish peroxidase‐conjugated secondary antibody (goat anti‐rabbit IgG H&L [Biotin] ab97049, 1:500), the VECTASTAIN ABC Kit (Vector Laboratories) and SIGMAFAST 3,3‐diaminobenzidine as substrate (Sigma, St. Louis, MO, USA). Sections were counter‐stained with haematoxylin.

### Real‐time polymerase chain reaction

Total RNA was isolated from tissues with TRIzol® (Invitrogen, Carlsbad, CA, USA), following the manufacturer's recommendations. The first strand of cDNA was generated from 2 μg of total RNA extracted, using a commercially available kit (High‐Capacity cDNA Reverse Transcription Kits, Invitrogen, Foster City, CA, USA). Polymerase chain reaction (PCR) amplification was performed in duplicates with SYBR Green PCR Master Mix (Applied Biosystems, Foster City, CA, USA) in the QuantStudio 12K Flex Real‐Time PCR (Applied Biosystems), adopting the primers listed in *Table* *1*. Gene expression was normalized to the ribosomal protein L27 (Rpl27) reference gene. Data were calculated using the 2^−ΔΔCT^ method and are presented as fold changes in gene expression relative to control samples.

**Table 1:** Body and tissue mass in control (C) and cachectic (T) animals

	T0	T7	T14	C14
Baseline			256.9 ± 6.86	267.12 ± 8.82
BM–TM (g)	266.3 ± 17.1	278.97 ± 8.79	296.61 ± 10.56*	328.2 ± 9.12
TM (g)		5.23 ± 2.35	19.19 ± 4.70	
Liver (g)	6.32 ± 0.72	8.60 ± 0.71	11.17 ± 1.56	11.03 ± 1.13
Soleus (g)			0.11 ± 0.02^#^	0.13 ± 0.01
Gastrocnemius (g)			1.63 ± 0.17*	2.0 ± 0.10
MEAT (g)	1.10 ± 0.56	1.47 ± 0.22	1.24 ± 0.34	1.60 ± 0.23
RPAT (g)	0.97 ± 0.4	2.11 ± 0.75	2.17 ± 0.75	2.79 ± 1.30
EAT (g)	1.74 ± 0.54	3.06 ± 0.78	3.26 ± 0.82	3.39 ± 0.85

*Note*: Values are expressed as mean ± SEM (*n* = 5). Abbreviations: BM–TM, body mass–tumour mass; EAT, epididymal adipose tissue; Abbreviations: MEAT, mesenteric adipose tissue; RPAT, retroperitoneal adipose tissue; TM, tumour mass.

* *P* < 0.01.

^#^ *P* = 0.051.

### Liver cytokine levels

Rat livers (0.05–0.08 g) were homogenized in radioimmunoprecipitation assay (RIPA) buffer (0.625% Nonidet P‐40, 0.625% sodium deoxycholate, 6.25‐mM sodium phosphate and 1‐mM ethylenediaminetetraacetic acid, at pH 7.4), containing a protease inhibitor cocktail (proteinase and phosphatase inhibitors, Roche). Homogenates were centrifuged at 15 000 × *g* for 30 min at 4°C. The supernatant was collected, and protein concentration was determined with a bicinchoninic acid assay (BCA) protein quantification kit (Pierce, Rockford, IL, USA), with bovine serum albumin (BSA) as a reference. Liver samples were assessed for the content interleukin‐6 (IL‐6) (DuoSet, DY506), tumour necrosis factor‐α (TNF‐α) (DuoSet, DY510), interleukin‐10 (IL‐10) (DuoSet, DY522) and interleukin‐1β (IL‐1β) (DuoSet, DY501). Protein concentration was measured by enzyme‐linked immunosorbent assay (ELISA) (DuoSet ELISA, R&D Systems, Minneapolis, MN, USA). Assay sensitivities were determined to be 8000 pg/mL in the range of 125–8000 pg/mL for IL‐6 and 4000 pg/mL in the range of 62.5–4000 pg/mL for TNF‐α, IL‐10 and IL‐1β, and the results were normalized to total protein. For IL‐1β measurement in the serum, we performed multiplex assay (MILLIPLEX® MAP Rat Adipokine Panel, RADPKMAG‐80K). All samples were run as duplicates, and the mean value was reported.

### Western blotting

Frozen livers were homogenized in RIPA buffer (0.625% Nonidet P‐40, 0.625% sodium deoxycholate, 6.25‐mM sodium phosphate and 1‐mM ethylenediaminetetraacetic acid, at pH 7.4, with proteinase and phosphatase inhibitors, Roche). Homogenates were centrifuged at 15 000 × *g* for 30 min at 4°C; the supernatant was collected, and protein concentrations were determined with a BCA protein quantification kit (Pierce, Rockford, IL, USA), with BSA as a reference. Samples containing 50‐mg protein were separated by electrophoresis in 10% Tricine–sodium dodecyl sulfate–polyacrylamide gel electrophoresis (SDS–PAGE). Proteins were then transferred to polyvinylidene difluoride (PVDF) membranes at 25 V for 40 min (Trans‐Blot Turbo Blotting System, Bio‐Rad) in transfer buffer, consisting of 20‐mM Tris, 150‐mM glycine and 10% methanol. PVDF membranes were then blocked in Tris‐buffered saline (TBS) containing 0.1% Tween 20 and 5% (w/v) non‐fat dry milk for 1 h. After three washes with TBS containing 0.1% Tween 20, the PVDF membranes were incubated with primary antibodies against phosphorylated c‐Jun N‐terminal kinase (p‐JNK) (Santa Cruz, SC‐6254 mouse), c‐Jun N‐terminal kinase (JNK) (Santa Cruz [D‐2], SC‐7345 mouse), toll‐like receptor 4 (TLR4) (Santa Cruz, SC‐30002 rabbit), IL‐1β (Santa Cruz, SC‐1251 goat) and glyceraldehyde 3‐phosphate dehydrogenase (GAPDH) (Santa Cruz, SC‐25778 rabbit). HRP‐conjugated secondary antibody anti‐rabbit (Cell Signaling #5127), anti‐mouse (Cell Signaling #7076) and anti‐goat (Abcam ab 6885‐1 UK) for 2 h at room temperature and bands were detected by enhanced chemiluminescence (Amersham, Little Chalfont, UK). The blots were stripped and incubated with anti‐GAPDH (Santa Cruz, SC‐25778 rabbit) as a loading control. Quantification of antigen–antibody complexes was performed by employing the ImageJ analysis software (http://rsb.info.nih.gov/ij/). Optical density units were given in pixels for fold target protein/control protein.

### Statistical analysis

Data are expressed as mean values and standard error of the mean. Differences among the experimental groups (T0, T7 and T14) were analysed with *GraphPad5 software* (GraphPad, San Diego, CA, USA), and statistical significance was determined by performing one‐way analysis of variance (ANOVA) with Tukey's post hoc test for comparison among groups. Body and tissue mass on Days 0 and 14 for control and tumour were analysed using Student's *t* test. For food consumption assessment, we analysed data by employing the two‐way ANOVA repeated analyses with Bonferroni's multiple comparison test. A value of *P* < 0.05 was considered significant.

## Results

**Figure 1:**
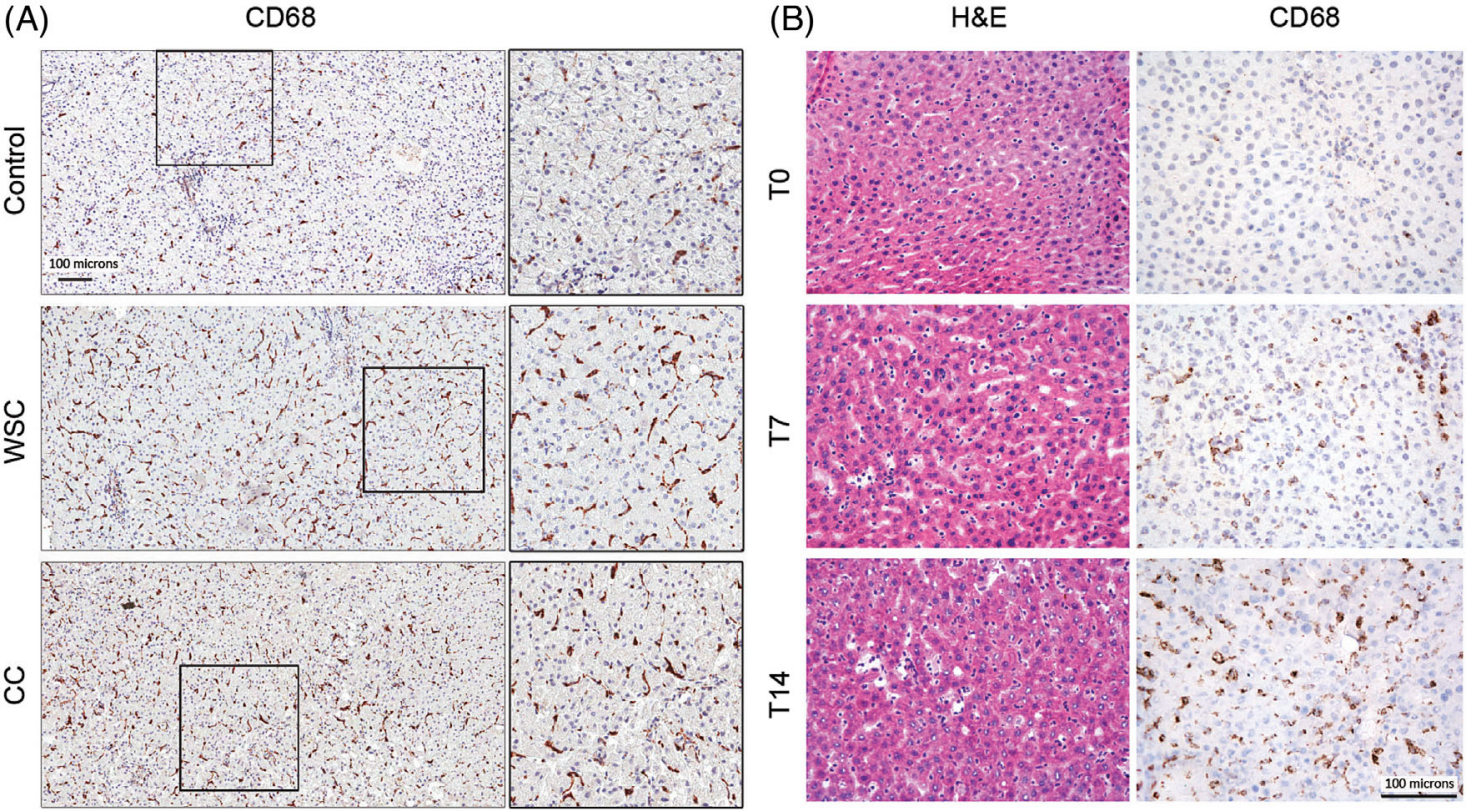
CD68^+^ cell infiltration in the liver of a cachectic patient. (A) Immunohistochemistry for CD68 in the liver of control, weight‐stable and cachectic cancer patients. ×20 and ×40 magnification. (B) H&E staining and immunohistochemistry for CD68 in rodent liver in T0, T7 and T14 after tumour injection. ×40 magnification.

In order to elucidate the mechanisms by which the liver contributes to systemic inflammation in cancer cachexia, we employed the Walker 256 model within a time‐course approach to address the progression of the changes (7 and 14 days after tumour injection). We examined cell infiltration and transcription factor activation during the development of cancer cachexia. Corroborating Martignoni et al.^12^ results, we also report CC patient liver to show a qualitative increase in CD68^−^ myeloid population, compared to WSC and with the control patient (*Figure* *1* *A*). The challenges imposed to obtain a sufficient number of biopsies from patients as to support quantitative analysis of liver inflammation in the scenario of cancer cachexia (especially when assessing temporal evolution) led us to employ the well‐characterized rodent W256 model, to the end of describing the molecular aspects of liver inflammation in cachexia. Fourteen days after tumour cell injection, experimental cachexia induced a 10.6% reduction in body weight gain (compared to age‐matched control animals) (*Table* *1*). The white adipose tissue did not show a significant change in T14 compared to C14; however, the muscle, specifically the gastrocnemius, showed reduced mass in T14 compared to C14, indicating cachexia‐associated atrophy (*Table* *1*). We also observed a reduction of food consumption after 8 days after tumour injection (*Figure* *S1*) as compared with the control group. In accordance with the literature, the reduction in food consumption is a synergetic effect on body wasting of cancer cachexia, but not its trigger.^15^ In addition, we observed a qualitative increase in the CD68^+^ myeloid population in the organ already after 7 days of the tumour cell injection, which was more pronounced on Day 14 after tumour injection (*Figure* *1* *B*).

**Figure 2:**
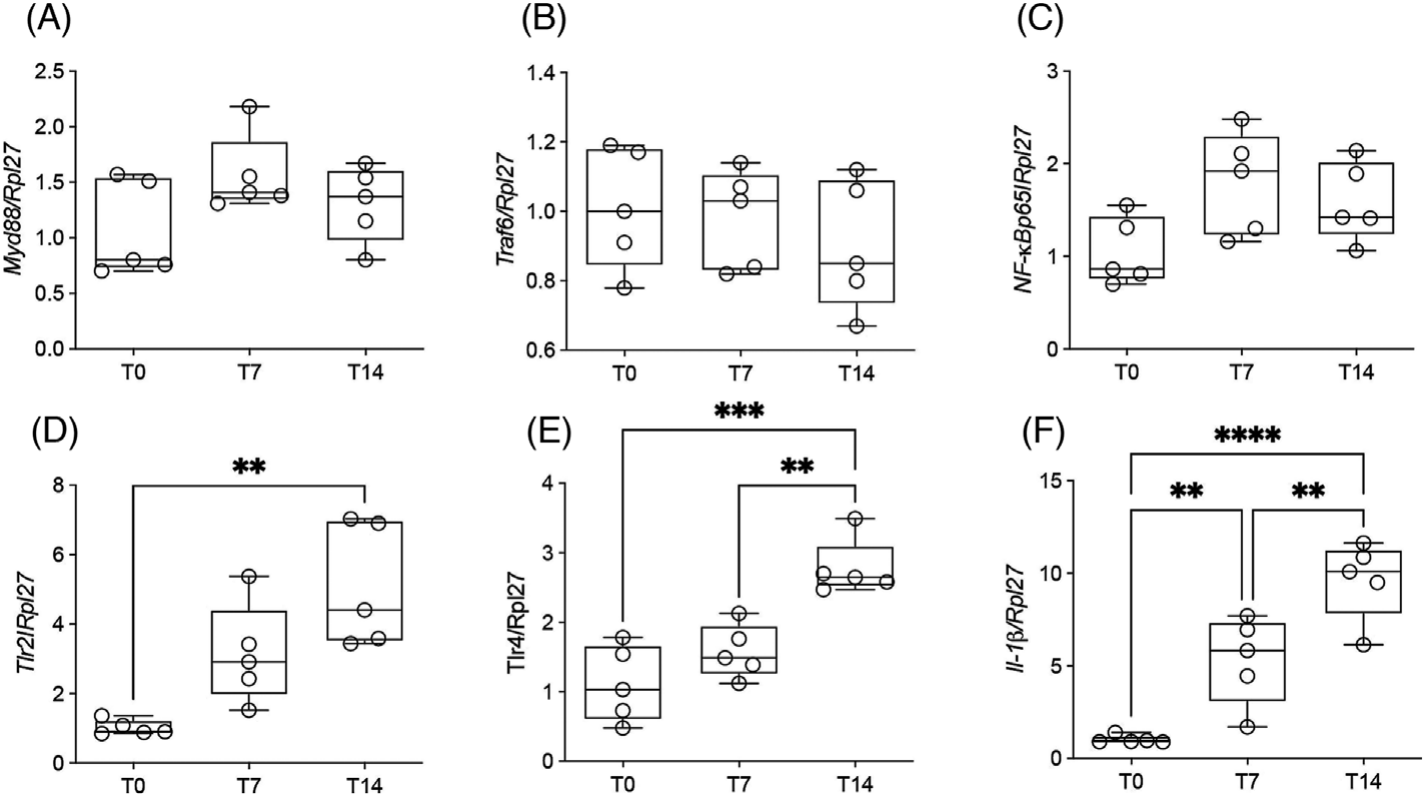
Gene expression of inflammatory pathways in the liver along cachexia progression. Values are expressed as box and whiskers min–max (*n* = 5). The liver was collected at three time points (T0, T7 and T14 after tumour injection). ^**^ *P* < 0.01. ^***^ *P* < 0.001. ^****^ *P* < 0.0001. (A) Gene expression of *Myd88*. (B) Gene expression of *Traf6*. (C) Gene expression of *NF‐κBp65*. (D) Gene expression of *Tlr2*. (E) Gene expression of *Tlr4*. (F) Gene expression of *IL‐1β*.

**Figure 3:**
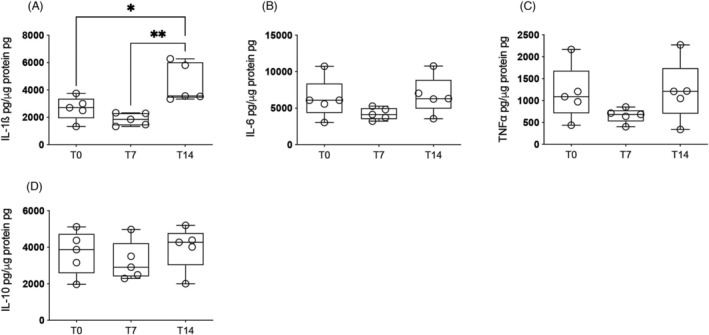
Inflammatory cytokine expression in rodent liver along the progression of cachexia. Values are expressed as box and whiskers min–max (*n* = 5). The liver was collected at three time points (T0, T7 and T14 after tumour injection). *P < 0.05. ^**^ *P* < 0.01. (A) Protein expression of total IL‐1β. (B) Protein expression of IL‐6. (C) Protein expression of TNF‐α. (D) Protein expression of IL‐10.

We performed gene expression analysis aiming at genes upstream of the NF‐κB pathway, such as myeloid differentiation primary response 88 (*Myd88*) and TNF receptor‐associated factor (*Traf6*), required for activation of NF‐κB, and also the subunit p65 of NF‐κB (RelA). None of these were statistically different for any of the time points analysed (*Figure* *2* *A*–*C*), suggesting that the NF‐κB pathway does not play a role in rodent hepatic inflammation. We found that *Tlr2* and *Tlr4* gene expression was increased on Day 14 (*Figure* *2* *D*,*E*), which could be associated with inflammation and the induction of various downstream proteins and activation of the inflammasome.^16^ In line with CD68^+^ myeloid population infiltration in the liver observed in the human CC sample, rodent samples and published data,^12^ we found gene expression for myeloid recruitment protein to be increased, *Ccl2*; in addition, markers of CD68^+^ myeloid population, *F4/80* and *Cd68*, also increased during cancer‐cachexia development (*Figure* *S2* *A*–*C*). *L‐1β* gene expression was higher on Day 7 and still increasing on Day 14 (*Figure* *2* *F*) and then accompanied by higher Il‐1β protein levels in the organ (*Figure* *3* *A*). A positive correlation was observed between *Ccl2*, *F4/80* and *Cd68* genes and the *IL‐1β* gene (*Figure* *S2* *D*–*F*), suggesting that CD68^+^ myeloid population infiltration is related to the increased *IL‐1β* expression. TLR4 can also induce the transcription activation of Il‐1β. Although Il‐1β was higher in the liver in terminal cachexia, the other cytokines examined, such as IL‐6, TNF‐α and IL‐10, were not significantly different to those observed in T0 (*Figure* *3* *B*–*D*).

**Figure 4:**
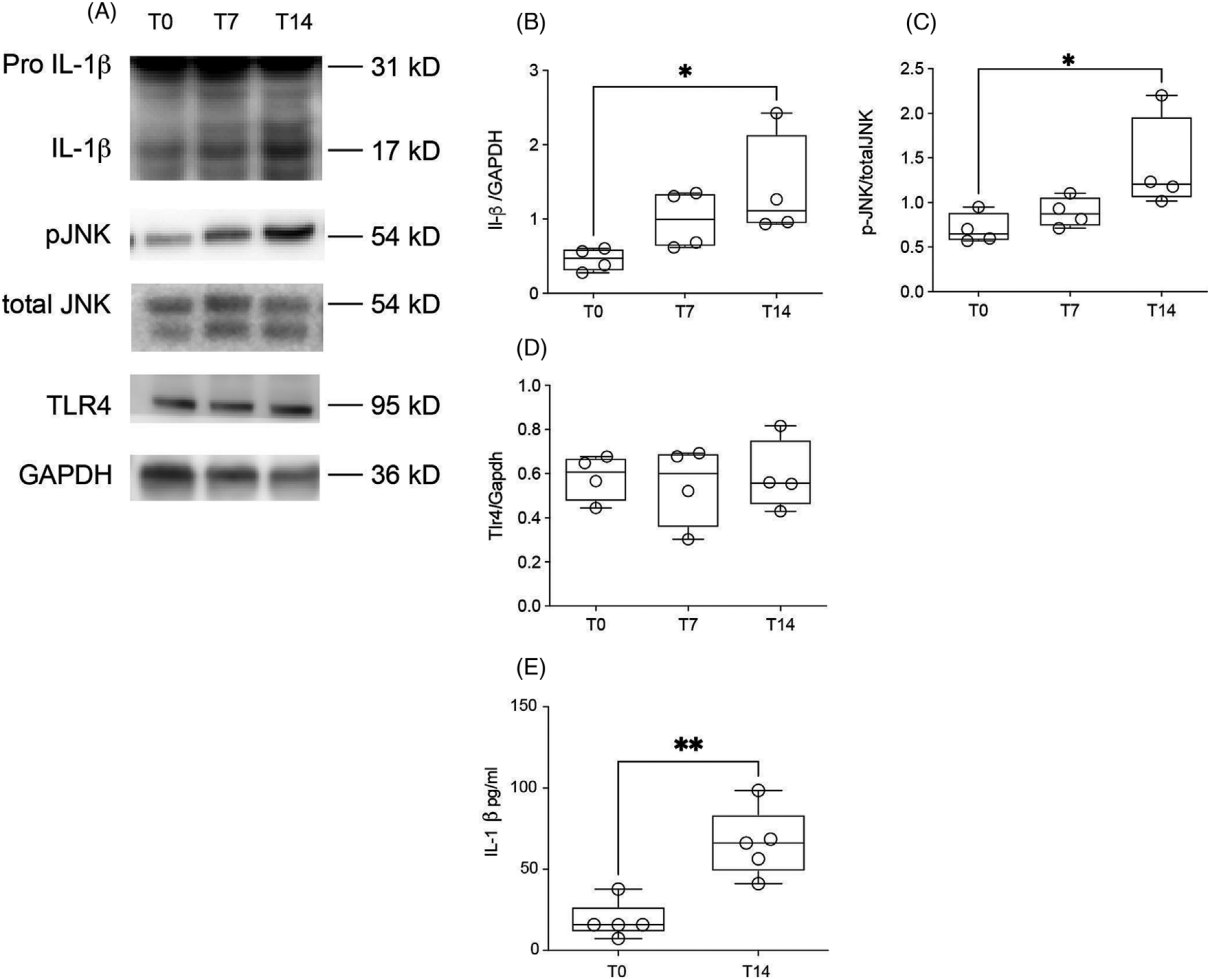
Inflammatory protein expression in rodent liver along cachexia progression. Values are expressed as box and whiskers min–max (*n* = 4). The liver was collected at three time points (T0, T7 and T14 after tumour injection). *P < 0.05. ^**^ *P* < 0.01. (A) Western blot images. (B) Protein expression of cleavage Il‐1β. (C) Protein expression of p‐JNK. (D) Protein expression of TLR4. (E) IL‐1β protein in the serum.

To determine whether IL‐1β protein content was translating into the activated form of the protein, we performed western blot for cleaved fraction detection, which showed increases on T14 (*Figure* *4* *B*). We searched for possible pathways that would induce transcription of the *IL‐1β* gene, having found that p‐JNK protein content was enhanced in T14 (*Figure* *4* *C*). Nevertheless, this transcription factor seems not to be activated by TLR4, because the latter was unchanged in the livers of cachectic rodents, as compared to controls (*Figure* *4* *D*).

**Figure 5:**
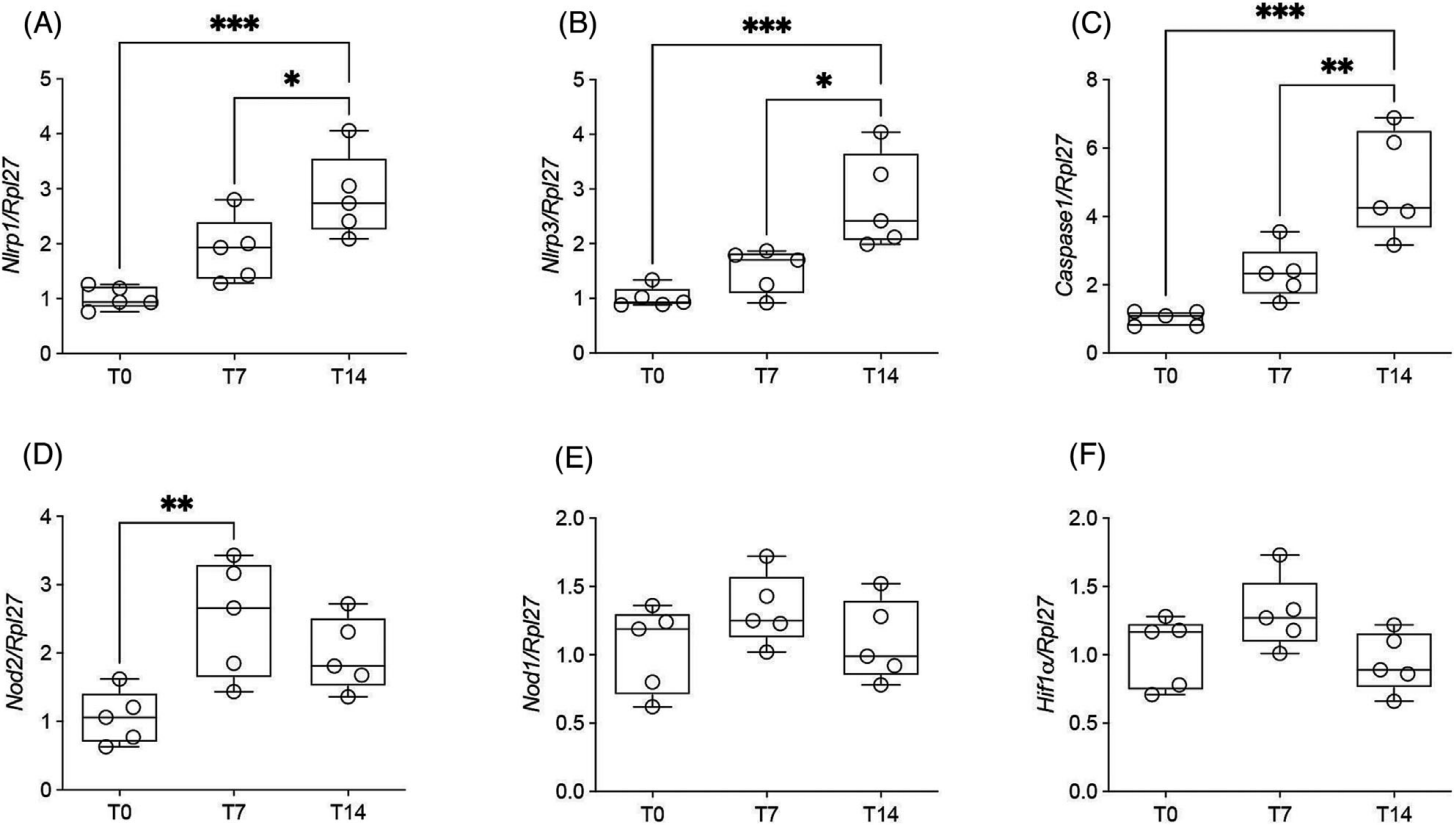
Gene expression of proteins of the inflammasome pathway in rodent liver along the progression of cachexia and circulating IL‐1β concentration. Values are expressed as box and whiskers min–max (*n* = 5). The liver was collected at three time points (T0, T7 and T14 after tumour injection). *P < 0.05. ^**^ *P* < 0.01. ^***^ *P* < 0.001. (A) Gene expression of *Nlrp1*. (B) Gene expression of *Nlrp3*. (C) Gene expression of *Caspase 1*. (D) Gene expression of *Nod2*. (E) Gene expression of *Nod1*. (F) Gene expression of *Hif‐1α*.

To confirm that the inflammasome pathway is playing a role in cancer cachexia, we assessed the gene expression of *Nlrp1* and *Nlrp3*, which were increased in T7 and T14 (*Figure* *5* *A*,*B*). We also observed that *Caspase 1* gene expression increased in terminal cachexia (*Figure* *5* *C*), whereas *Nod2* gene expression was increased only on T7, returning to basal levels in T14 (*Figure* *5* *D*), whereas *Nod1* and *Hif‐1α* gene expression did not change during the development of cachexia (*Figure* *5* *E*,*F*). To assess whether augmented IL‐1β production in the liver reflected systemically, we performed ELISA for IL‐1β protein content in the serum, and indeed, the cytokine was increased in T14 compared to T0 (*Figure* *4* *E*).

## Discussion

Cancer cachexia is a complex, intricate and multifactorial syndrome, encompassing a diversity of alterations that markedly decrease survival and compromise quality of life. Many studies have described the complex interplay between the tumour and the host's tissues in the aetiology of chronic systemic inflammation, which is associated with many of the symptoms of cachexia.^2^ We herein present results that suggest that the liver contributes to chronic systemic inflammation during the development and progression of cancer cachexia in rats bearing the Walker 256 carcinosarcoma, a broadly studied model of cancer cachexia.^8^, ^17^, ^18^

The liver parenchyma and stroma are comprised by many different cell types, all of which have a role in the secretion of cytokines and chemokines in health and disease.^19^ During chronic disease, the number of infiltrating cells in the organ, especially that of monocytes, may be greatly increased, as reported for obesity and diabetes.^20^, ^21^ In the present study, we found a linear increase in the CD68^+^ myeloid population, paralleling the progression of cachexia, which was endorsed by the finding of increased detection of specific receptors (*F4/80* and *Cd68*) and chemokine (*Ccl2*) genes of CD68^+^ population. Previous to our study, Martignoni et al.^12^ described CD68^+^ myeloid population infiltration in the liver of cachectic pancreatic cancer patients. The same authors showed an increase in IL‐6 protein expression, as detected by immunohistochemistry in the liver of the patients. Nevertheless, they failed to produce evidence regarding Il‐1β protein expression.

Mice under high‐fat diet show liver inflammation.^22^ The gene expression levels of TNF‐α, IL‐1β and IL‐6 are concomitantly increased, and one such inflammatory reaction has been suggested to play a major contribution to hepatic fibrosis and disruption of lipid metabolism.^23^, ^24^ A previous study by our group reported that the livers of cachectic rodents suffer impairment of long‐chain fatty acid oxidation, along with disruption of very‐low‐density lipoprotein secretion, leading to steatosis.^25^

Infiltration of immune cells in cachexia does not represent a novel finding as, for instance, the white adipose of both cachectic rodents and cancer patients shows the increased density of inflammatory cells^26^, ^27^, ^28^ from which secreted active mediators trigger fibrosis.^26^ That may be in fact the reason why when analysed as bulk wet weight, the mass of the adipose depots did not change in the present experiment. Marked immune cell infiltration, in parallel to oedema and fibrosis (all previously reported findings by our group, in both human and rodent adipose tissue) in cachexia,^29^, ^30^ may account for the masking of triacylglyceride loss.

In the present study, we describe an increase of the CD68^+^ myeloid population also in the liver, across the progression of cachexia, which may be the cause for increased IL‐1β protein secretion that can be detected in the circulation. Thus, as demonstrated for the white adipose tissue, the liver may contribute to local and systemic inflammation in cancer cachexia.

The liver is a central organ in the management of intermediary metabolism. In some diseases, as in cachexia, its metabolism is disrupted, with major consequences to the host's metabolic control and substrate utilization.^6^, ^25^ We have also previously described that treatment with indomethacin, decreasing the production of PGE_2_, another relevant inflammatory factor, in cachectic rats, ameliorates metabolic parameters in the organ and partly recovers cachexia‐induced wasting.^31^ It seems thus clear that the combination of metabolic disruption and inflammation that is systemic in cachexia is also present at the organ/tissue level. The inflammatory reaction we herein report, with increased hepatic IL‐1β gene and protein expression, concomitant to up‐regulation of the inflammasome pathway and with JNK protein activation, seems to be correlated with CD68^+^ myeloid population infiltration that we found. JNK is considered a potential inflammatory mediator of metabolic changes in the liver.^23^ JNK knockout mice consuming a high‐fat diet showed improvement in hepatic fatty acid oxidation, compared to wild‐type (WT) mice under the same diet regimen. This strongly suggests that JNK plays a role in the modulation of fatty acid oxidation and in the induction of steatosis and fibrosis.^23^

IL‐1β is considered a strong pro‐inflammatory cytokine and has been shown to play a role in obesity‐induced liver steatosis by up‐regulating gene expression of lipogenic enzymes.^32^ The activity of IL‐1β is regulated by the inflammasome complex pathway, and this aspect has been studied in many different diseases.^33^, ^34^ The inflammasome complex activates IL‐1β by cleavage of the immature form into mature IL‐1β, which, when released, may bind to the IL‐1 receptor at local or distant sites, inducing activation of the IL‐1β cascade.^35^ We failed to find in the literature studies showing the role of the inflammasome pathway in the cachectic patient's liver. However, it has been reported that the IL‐1β content in the serum of cachectic patients is increased. As found in the adopted rodent model, the levels of IL‐1 β in the serum and also in the liver are increased in cachexia. Moreover, this observation was accompanied by the identification of an up‐regulation of genes taking part in the inflammasome complex in the organ along cancer‐cachexia progression. We may hence assume that the higher inflammasome activity in the liver is leading to higher levels of systemic IL‐1β and contributing to induction of systemic inflammation.

It is possible to speculate that inflammation at both the systemic and organ levels subsidizes metabolic changes, as suppression of inflammatory pathways reduces wasting. Nevertheless, it is important to comment on two aspects: Firstly, at the moment, no teleological relationship has been fully proven, as it could be inflammation causing metabolic disruption, but one cannot discard that the opposite, metabolic changes inducing inflammation, or still concomitant action in the scenario of cachexia. The second relevant point is that inflammation presents many faces and a plethora of variations. We show clear evidence that, differently from the reported to the adipose tissue, in which both NF‐κB and inflammasome pathways are activated in cancer cachexia,^36^, ^37^ in the liver, the inflammasome pathway seems to be the main villain, as we found no changes in regard to proteins taking part in the NF‐κB via. That could initiate discussion on organ‐specific drug targeting, aiming at mitigating the effects of inflammation in wasting and metabolic disruption at specific sites, within a precision medicine approach.

Limitations of this study should be acknowledged. Human sample availability was the main limitation for performing statistical analysis as well as different techniques, such as real time–quantitative PCR (RT‐qPCR), ELISA and western blot, to validate the finding for human cachexia. We stress the difficulties in obtaining human liver samples, in the absence of hepatic metastases. In addition, having a pair‐fed group in the animal arm would reinforce the results that those changes in the liver are due to cancer‐cachexia development instead of a reflex of reduced food consumption. Even so, we believe the results to have relevance, in the face of constrictions in the obtainment of human liver biopsies and the original aspect of demonstrating the involvement of the inflammasome pathway.

**Figure 6:**
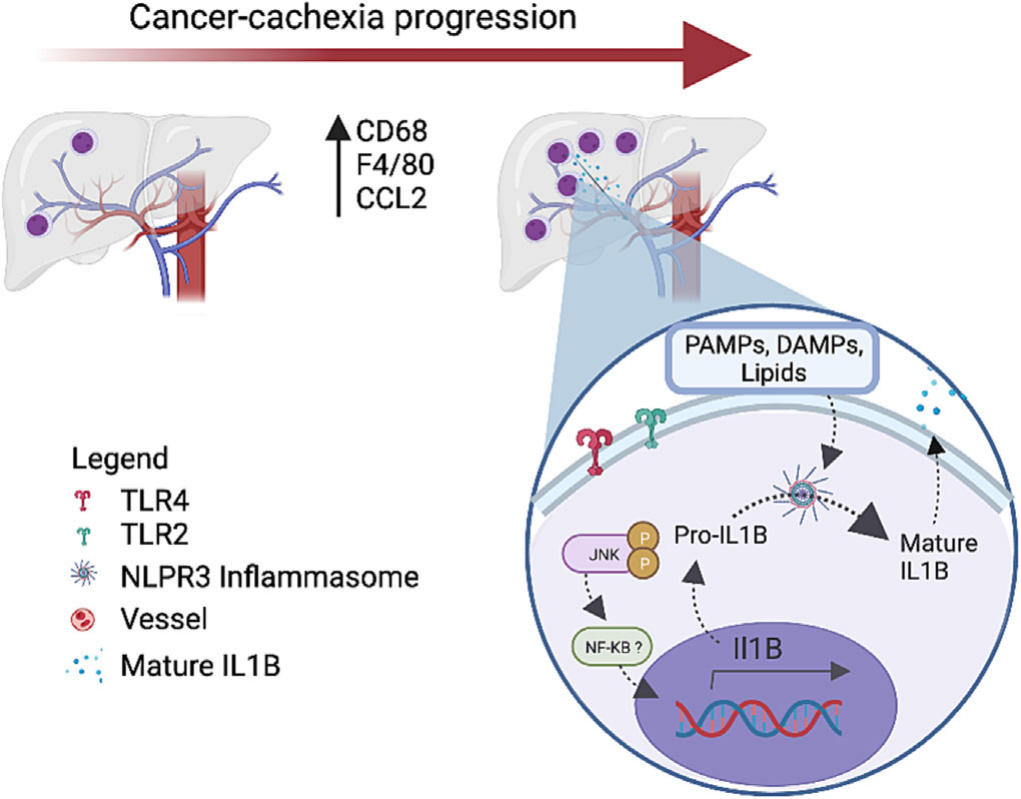
The summary of the main results from this paper shows the contribution of the liver to systemic inflammation by the increase of pro‐inflammatory cytokine (IL‐1β) and inflammasome pathway in the liver during cancer cachexia.

In conclusion, our results demonstrate for the first time, to the best of our knowledge, that the inflammasome pathway plays a role in the liver in cachexia, eliciting IL‐1β maturation. The reported activation is suggested to be associated with the presence of an increased CD68^+^ myeloid population, also reported here in human cachexia, in a time‐dependent manner; the mechanism summary is illustrated in *Figure* *6*. Targeting these inflammasome‐associated changes in the pre‐cachectic stage could pose as a new therapeutic possibility to prevent disease progression and improve patient outcome and survival rate.

## Funding

This study was supported by Fundação de Amparo à Pesquisa do Estado de São Paulo (FAPESP; 2012/50079‐0 and 2020/07765‐6).

## Conflict of interest statement

The authors declare that the research was conducted in the absence of any commercial or financial relationships that could be construed as a potential conflict of interest.

## Supporting information


**Figure S1.** Food consumption on day 14 of Control (C14) and Tumour‐bearing (T14) animals). A‐ Food consumption assessment was performed every day and the data are expressed as mean ± SD. ***p < 0.001.

**Figure S2.** Correlation between Il‐1β gene expression with genes related to CD68+ myeloid population infiltration. Values are expressed as Box and Whiskers min‐max (*n* = 5). The liver was collected at time points (T0, T7, and T14 post‐tumour injection). *p < 0.05; **p < 0.01. A‐ gene expression of F4/80. B‐ gene expression of CD68. C‐ gene expression of Ccl2. Pearson correlation coefficient (R) was adopted to determine the relationship between Il1β gene expression to genes markers for CD68+ myeloid population. D‐ Il‐1β and F4/80. E‐ Il‐1β and Cd68. F‐ Il‐1β and Ccl2. P‐value of <5% was considered statistically significant.
