# Supplementary figures and images for: Cachexia causes time‐dependent activation of the inflammasome in the liver
Source: J Cachexia Sarcopenia Muscle. 2023 May 12;14(4):1621–30. doi: 10.1002/jcsm.13236 (PMC10401524; doi:10.1002/jcsm.13236)

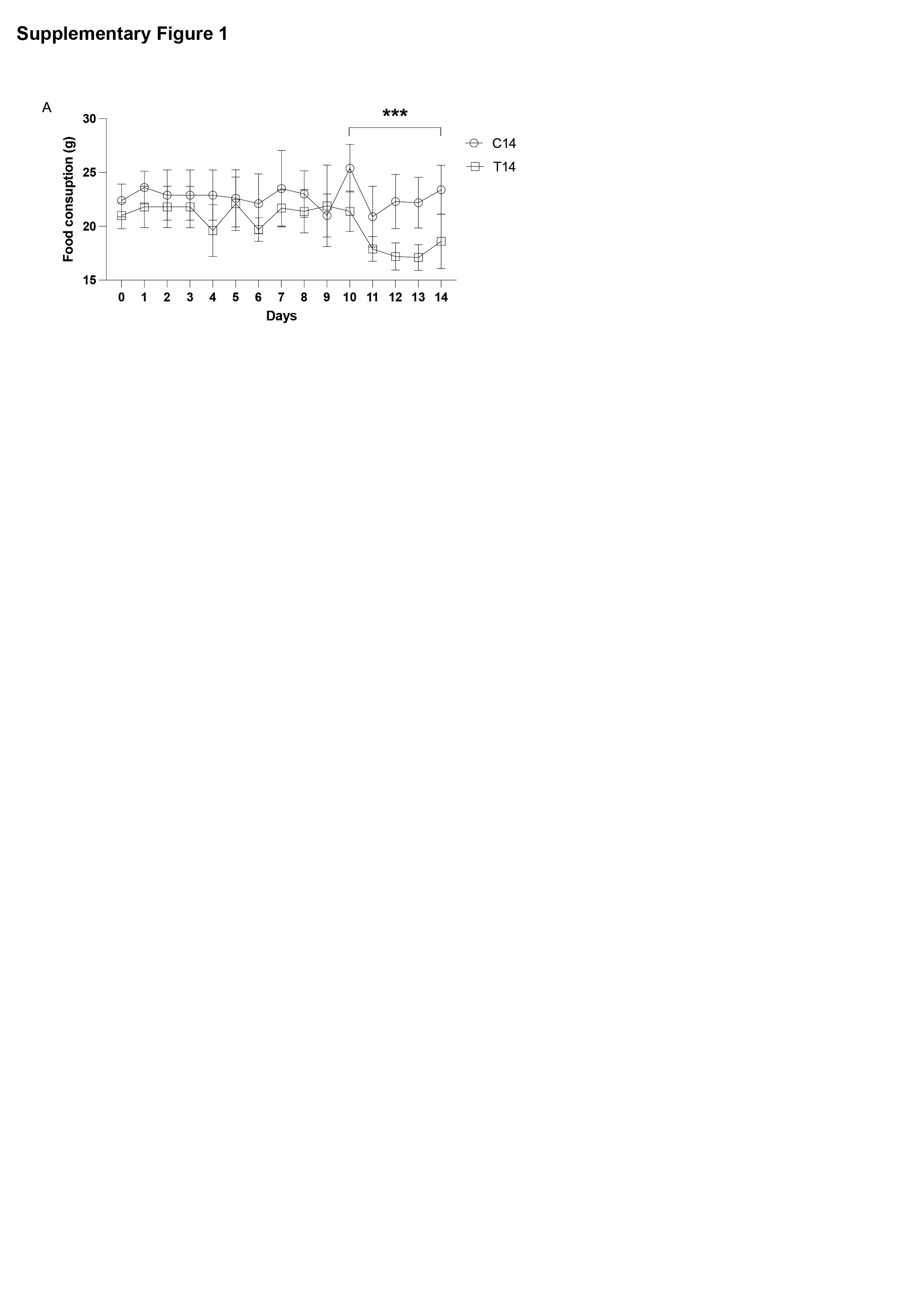

Supplement: Supplementary file 1 — Figure S1. Food consumption on day 14 of Control (C14) and Tumour‐bearing (T14) animals). A‐ Food consumption assessment was performed every day and the data are expressed as mean ± SD. ***p < 0.001. [file JCSM-14-1621-s001.jpg]

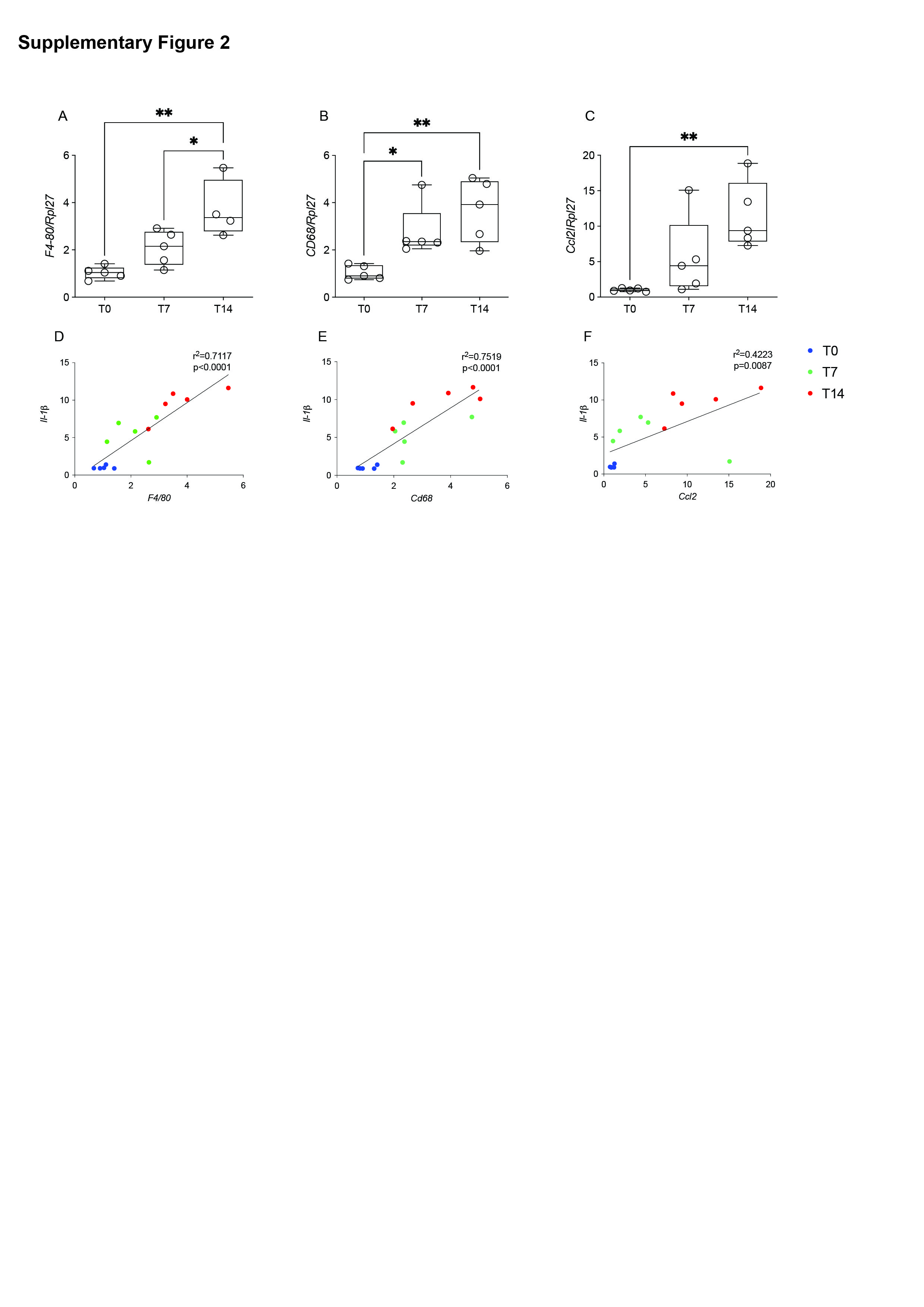

Supplement: Supplementary file 2 — Figure S2. Correlation between Il‐1β gene expression with genes related to CD68+ myeloid population infiltration. Values are expressed as Box and Whiskers min‐max (n = 5). The liver was collected at time points (T0, T7, and T14 post‐tumour injection). *p < 0.05; **p < 0.01. A‐ gene expression of F4/80. B‐ gene expression of CD68. C‐ gene expression of Ccl2. Pearson correlation coefficient (R) was adopted to determine the relationship between Il1β gene expression to genes markers for CD68+ myeloid population. D‐ Il‐1β and F4/80. E‐ Il‐1β and Cd68. F‐ Il‐1β and Ccl2. P‐value of <5% was considered statistically significant. [file JCSM-14-1621-s002.jpg]
